# *In vitro* Evidence of Human Immune Responsiveness Shows the Improved Potential of a Recombinant BCG Strain for Bladder Cancer Treatment

**DOI:** 10.3389/fimmu.2019.01460

**Published:** 2019-06-26

**Authors:** Dunia Rodriguez, Cibelly Goulart, Ana C. Pagliarone, Eliane P. Silva, Priscila S. Cunegundes, Ivan P. Nascimento, Ricardo C. Borra, Waldely O. Dias, Aldo Tagliabue, Diana Boraschi, Luciana C. C. Leite

**Affiliations:** ^1^Laboratório de Desenvolvimento de Vacinas, Instituto Butantan, São Paulo, Brazil; ^2^Programa de Pós-Graduação Interunidades em Biotecnologia USP-I.Butantan-IPT, São Paulo, Brazil; ^3^Laboratório de Imunologia Aplicada, Departamento de Genética e Evolução, Universidade Federal de São Carlos, São Carlos, Brazil; ^4^Institute of Genetic and Biomedical Research, National Research Council, Cagliari, Italy; ^5^Institute of Protein Biochemistry, National Research Council, Naples, Italy

**Keywords:** recombinant BCG, bladder cancer, human immune cells, immunotherapy, adjuvant, CD4 T cells, cytokines

## Abstract

The live attenuated mycobacterial strain BCG, in use as vaccine against tuberculosis, is considered the gold standard for primary therapy of carcinoma *in situ* of the bladder. Despite its limitations, to date it has not been surpassed by any other treatment. Our group has developed a recombinant BCG strain expressing the detoxified S1 pertussis toxin (rBCG-S1PT) that proved more effective than wild type BCG (WT-BCG) in increasing survival time in an experimental mouse model of bladder cancer, due to the well-known adjuvant properties of pertussis toxin. Here, we investigated the capacity of rBCG-S1PT to stimulate human immune responses, in comparison to WT-BCG, using an *in vitro* stimulation assay based on human whole blood cells that allows for a comprehensive evaluation of leukocyte activation. Blood leukocytes stimulated with rBCG-S1PT produced increased levels of IL-6, IL-8, and IL-10 as compared to WT-BCG, but comparable levels of IL-1β, IL-2, IFN-γ, and TNF-α. Stimulation of blood cells with the recombinant BCG strain also enhanced the expression of CD25 and CD69 on human CD4^+^ T cells. PBMC stimulated with rBCG-S1PT induced higher cytotoxicity to MB49 bladder cancer cells than WT-BCG-stimulated PBMC. These results suggest that the rBCG-S1PT strain is able to activate an immune response in human leukocytes that is higher than that induced by WT-BCG for parameters linked to better prognosis in bladder cancer (regulation of immune and early inflammatory responses), while fully comparable to WT-BCG for classical inflammatory parameters. This establishes rBCG-S1PT as a new highly effective candidate as immunotherapeutic agent against bladder cancer.

## Introduction

The Bacille-Calmette-Guérin (BCG) is a live anti-tuberculosis vaccine, which has been administered to more than 3 billion individuals worldwide over 80 years (1–3). BCG is also used as immunotherapeutic treatment of non-muscle invasive bladder cancer (NMIBC). After over 40 years of use, BCG is still the gold standard for bladder cancer at early stage (4), by decreasing disease progression and the risk of recurrence (5). As mechanism of anti-tumor activity, BCG instilled into the bladder induces a local inflammatory immune response with influx of granulocytes and mononuclear cells and a potent production of inflammatory and Th1 cytokines, resulting in the activation of a significant anti-tumor response (6, 7). However, BCG immunotherapy has limitations, as ~30% of patients do not respond to BCG, and 50% recur after BCG therapy (8, 9). In an attempt to increase treatment efficacy, different strains of BCG have been used as immunotherapy, as yet without satisfactory results (10, 11).

Several recombinant BCG (rBCG) strains have been constructed as vaccine candidates for more efficient protection against tuberculosis (12) and for new vaccines against viruses, bacteria, and parasites (13, 14). Other rBCG strains have also been constructed, expressing inflammatory or Th1 cytokines such as IL-2, IL-12, IL-18, IFN-α, and IFN-γ, as potentially improved immunotherapeutic tools for bladder cancer treatment (15, 16). A rBCG strain expressing IFN-γ specifically increased MHC class I molecules in the bladder cancer cell line MB49 *in vitro* and could prolong survival in mice with murine orthotopic bladder cancer as compared to BCG treatment (17). Another rBCG strain, which expresses IFN-α, induced increased production of Th1 cytokines, and enhanced cytotoxicity by peripheral blood mononuclear cells (PBMC) against bladder cancer cell lines *in vitro* (18). Our group has constructed rBCG strains expressing toxin derivatives (19, 20), including the genetically detoxified S1 subunit of Pertussis Toxin-9K/129G (S1PT) (21). The rBCG-S1PT strain was obtained from a BCG Moreau background, the strain used in Brazil for both TB vaccination and bladder cancer immunotherapy (22, 23). Auxotrophic complementation allowed stable *in vivo* expression of the S1PT protein, and the new strain induced a specific cellular immune response that effectively protects neonate mice from *B. pertussis* challenge (24). Based on the well-known adjuvant properties of PT, i.e., its capacity to induce non-specific immune/inflammatory activation, we have evaluated the rBCG-S1PT strain for the immunotherapy of bladder cancer in a murine model, in comparison with wild type BCG (WT-BCG). That study demonstrated that the S1PT-expressing BCG strain decreased more effectively bladder weight and induced a highly significant increase in the survival of mice as compared with WT-BCG (25, 26). As this recombinant BCG strain is being prepared for evaluation in clinical trials as improved treatment of bladder cancer, we are providing here data in support of its improved immunotherapeutic performance by examining its capacity to induce immune activation of human cells *in vitro*.

## Materials and Methods

### Subjects and Blood Collection

Thirty-nine healthy adults (19 males and 20 females, aged 20–65 years) were recruited at the Hospital Universitário, Universidade de São Paulo. Clinical history (obtained in a questionnaire) and blood counts and serological parameters were used to confirm the health conditions of the participants. Thirty-four participants reported vaccination with live BCG at birth, while five were not vaccinated. Ten milliliters of blood were collected into heparinized tubes. The study protocol was approved by the Comitê de Ética/Pesquisa Hospital Universitário/USP (CEP-HU/USP) 728.275.

### BCG Strains

The rBCG-S1PT strains were constructed based on a BCG Moreau background (24). The BCG Moreau strain is one of the early strains (together with BCG Russia and Japan) as identified by genomic studies, while late strains include BCG Pasteur and Danish (27). Several studies suggest that early BCG vaccines may induce superior immune responses to the widely used late strains (27). The strain used here is a complemented auxotrophic recombinant BCG (24). The BCG Moreau strain and rBCG-S1PT were grown in Middlebrook 7H9 medium with albumin dextrose-catalase enrichment at 37°C with 5% CO_2_ using stationary tissue culture flasks. Bacteria were harvested by centrifugation, washed and stored in aliquots at −80°C. Aliquots were thawed and colony forming units (CFU) were determined before use by plating onto Middlebrook 7H10 medium with oleic albumin dextrose-catalase enrichment.

### Whole Blood Assay

Whole blood stimulation (28) was performed by diluting 250 μL of fresh heparinized human whole blood (containing an average of 1.8 × 10^6^ nucleated cells) in 750 μL of RPMI-1640 medium (GIBCO®, Life Technologies, Paisley, UK) in the presence of BCG or rBCG-S1PT (10^5^ CFU/mL) in 1.5 mL polypropylene tubes. Thus, the multiplicity of infection (MOI) obtained was approximately 0.1. Negative controls did not contain mycobacteria. Positive controls received 2.5 ng/mL LPS (from *E. coli* O55:B5; Sigma-Aldrich Inc., St. Louis, MO). Tubes were tightly capped, mixed by inversion, and incubated at 37°C for 24 or 48 h. An aliquot of 100 μL was taken for cell staining and phenotyping. The remaining cells in 0.9 mL were lysed with 100 μL Triton-X (1% final concentration), and samples frozen at −80°C for cytokine measurement.

### Cytokine Measurement

Frozen samples were thawed, centrifuged, and the supernatants assayed for inflammation-related cytokines at 24 h (IL-1β, IL-6, IL-8, IL-10, IL-12p70, TNF-α) and Th1/Th2/Th17-related cytokines at 48 h (IL-2, IL-4, IL-6, IL-10, TNF-α, IFN-γ, IL-17A) with Cytometric Bead Array kits (CBA; BD Biosciences, San Jose, CA), as per the manufacturer's instructions. The assays' lower limits of detection were between 2.6 and 18.9 pg/mL, depending on the cytokine, and the higher limit was 5,000 pg/mL. All samples were tested undiluted and, if values were above threshold, were diluted and retested. Samples below the lower threshold limit were considered as zero. Testing of some cytokines at both time points demonstrated that the majority/totality of production occurred within the first 24 h (Supplementary Figure 1). Values are expressed as pg or ng cytokines/mL of blood.

### Cell Phenotypic Analysis

Phenotypic analysis was performed after 48 h. Cells were treated with 2 μM EDTA (Sigma Aldrich Inc.) for 10 min and washed with PBS, then incubated for 30 min at room temperature with the following conjugated mAbs: CD3-APC-H7, CD4-PECy-5, CD25-BV510, and CD69-FITC. After incubation, red blood cells were lysed using 2 mL FACS lysing solution, and flow cytometric acquisition was performed with a FACS Canto II after optimization of settings using the cytometer setup and tracking beads. Instrument and reagents were from BD Bioscience. Data were analyzed using FlowJo version 7.6.5. No differences in the proportion of dead cells were evident between treatments and between treatments and controls, by scattering analysis.

### Cytotoxicity Assay

MB49-GFP cells (mouse bladder carcinoma cell line MB49 cells modified to constitutively express green fluorescent protein, GFP) were maintained in RPMI-1640 medium containing 10% heat-inactivated fetal bovine serum at 37°C in humidified air with 5% CO_2_ (25). The MB49-GFP cells were detached by trypsin treatment, seeded in 96-well flat-bottom microplates at 3.5 × 10^4^ cell/well, and incubated for 24 h until confluent. Human PBMC were isolated from heparinized blood samples with standard procedures using Ficoll-Paque (GE Healthcare Bio-Science AB, Uppsala, Sweden). PBMC were mixed with WT-BCG or rBCG-S1PT at MOI 0.1 and immediately added to MB49-GFP cell cultures at the initial PBMC to tumor cell ratios of 12.5:1, 25:1 and 50:1. After an overnight incubation, the plates were washed with sterile PBS twice, the MB49-GFP cells were detached by trypsinization (50 μL/well, 4 min), collected by centrifugation, resuspended in 50 μL/well of ice-cold RPMI-1640 and counted in a hemacytometer with a fluorescence microscope (Nikon E200).

Percent cytotoxicity (which in this assay measures the sum of cytolytic and cytostatic effects) was expressed by taking as 0 the number of tumor cells in the absence of PBMC and BCG, and 100% the absence of tumor cells. Neither WT-BCG not rBCG-S1PT at the concentrations used had a direct cytotoxic effect on tumor cells.

### Statistical Analysis

Statistical analysis was performed with the GraphPad Prism 6.02 software package (GraphPad software, San Diego, CA, USA). Analysis on cytokine levels and cell phenotypes were performed using a one-way analysis of variance (ANOVA) with a Bonferroni's multiple-comparison test, and the cytotoxicity assay using Student's *t*-test. Differences between groups with *p* ≤ 0.05 were considered significant.

## Results

### rBCG-S1PT Induces Cytokine Production in Human Blood Cells

**Figure 1:**
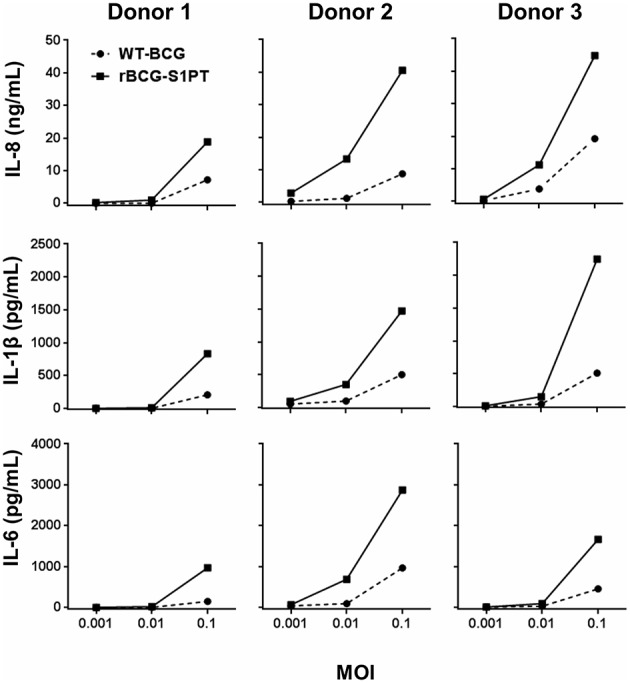
Dose-response of WT-BCG and rBCG-S1PT in activating cytokine production by human blood cells *in vitro*. Blood cells from three individual donors (Donors 1 and 2 had been vaccinated with BCG, Donor 3 was non-vaccinated) were exposed for 24 h to increasing MOI of WT-BCG or rBCG-S1PT. Cytokines were measured with a CBA assay and expressed as cytokine concentration /mL blood. Spontaneous release by non-stimulated cells was subtracted. This was 0.8–1.6 ng/mL for IL-8, 24–92 pg/mL for IL-1β, and 20–89 pg/mL for IL-6.

Preliminary experiments were run for selecting the WT-BCG and rBCG-S1PT MOI to use in the study. Based on literature data suggesting a MOI of 0.1 as optimal (29), we set up dose-response experiments with three donors (two vaccinated with BCG and one non-vaccinated), in which we compared the ability of WT-BCG and rBCG-S1PT to induce cytokine production by using a rapid and very efficient assay on whole blood, which assesses the global response of blood cells to exogenous stimuli (28). Cytokines assessed were the chemokine IL-8 and the inflammatory cytokines IL-1β and IL-6. MOI used were 0.1 and below, in an attempt to identify differences between the two BCG types in the perspective of dose reduction in future therapeutic approaches. The results in Figure 1 show that for both BCG strains the MOI 0.1 was the concentration that stimulated a substantial reaction in all donors. The response to rBCG-S1PT was higher than that to WT-BCG at this MOI and, for Donor 2 (all cytokines) and Donor 3 (IL-8), also at MOI 0.01. Minimal or no reaction was observed at MOI 0.001 for either BCG strain.

**Figure 2:**
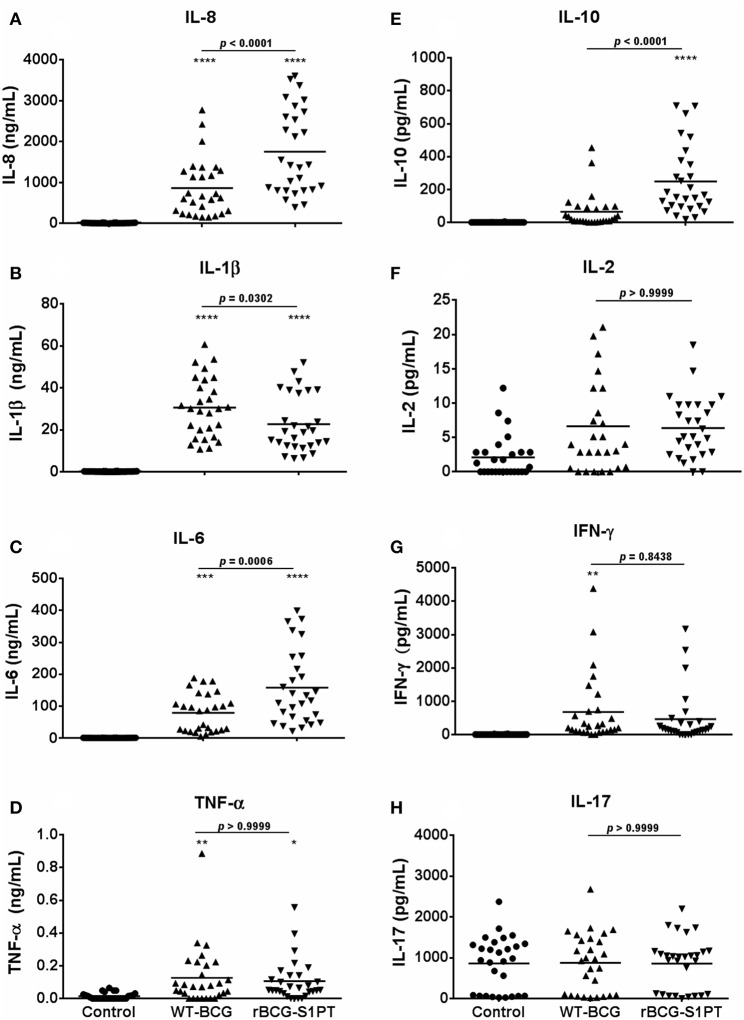
Production of cytokines by blood cells from healthy adults stimulated with WT-BCG or rBCG-S1PT. Blood cells were stimulated with WT-BCG or rBCG-S1PT and cytokines were measured with a CBA array. Data at 24 h are reported for the early inflammation-related cytokines IL-8 **(A)**, IL-1β **(B)**, IL-6 **(C)**, and TNF-α **(D)**, while results at 48 h are shown for the T-related cytokines IL-10 **(E)**, IL-2 **(F)**, IFN-γ **(G)**, and IL-17A **(H)**. Values represent the concentration of cytokines per mL blood. Negative controls are non-stimulated cells. Positive controls (cells stimulated with LPS) were: IL-8, 133.40 ± 13.63 ng/mL; IL-1β, 13.45 ± 1.28 ng/mL; IL-6, 80.11 ± 12.30 ng/mL; TNF-α, 0.16 ± 0.04 ng/mL; IL-10, 95.04 ± 17.61 pg/mL; IL-2, 3.58 ± 0.83 pg/mL; IFN-γ, 798.21 ± 187.08 pg/mL; and IL-17, 1004.74 ± 143.52 pg/mL. ****p < 0.0001, ***p < 0.001, **p < 0.01, and *p < 0.05 vs. negative controls. The *p* value for the difference between BCG groups is indicated over the bar.

Once chosen the MOI, we have tested the human response to BCG strains more extensively, using the same assay on whole blood cells. Since effector cells in a localized tissue inflammation are recruited from blood, assessing human blood cell reaction to BCG is a relevant way to evaluate local immune/inflammatory reactivity. Human blood from healthy donors was exposed *in vitro* to WT-BCG or rBCG-S1PT, and immune reactivity assessed after 24 and 48 h. The results reported in Figure 2 show that rBCG-S1PT induces a similar cytokine profile as WT-BCG, i.e., significant production of IL-8, IL-1β, IL-6, TNF-α, IL-10, and IL-2. For IFN-γ, only production induced by WT-BCG reached statistical significance, while that induced by rBCG-S1PT showed a tendency to increase that was not significant (*p* = 0.06) by ANOVA, although it was significant by the Student's *t* test (not shown). There was no significant production of IL-4 or IL-12 in response to either BCG strain (not shown), while IL-17 was produced constitutively and was not increased upon stimulation with BCG (Figure 2H). Notably, rBCG-S1PT was able to stimulate a significantly higher production of IL-8, IL-6, and IL-10 when compared to WT-BCG (Figures 2A,C,E). While production of inflammatory cytokines (IL-8, IL-1β) was generally examined at 24 h, and that of T-related cytokines (IL-2, IFN-γ, IL-17) at 48 h, the production of IL-6, TNF-α, and IL-10 was evaluated at both 24 and 48 h (Supplementary Figure 1). The cytokine profiles (including differences between recombinant and wild type BCG) were comparable between the two time points, although their level was lower at 48 h. This suggests that for these cytokines the production mainly occurred in the first 24 h.

By examining data in Figures 1, 2, a great variability in the individual response to BCG can be observed both in the amount of cytokine produced and reactivity to the different strains. Indeed, the response to BCG varies substantially among donors. As an example, IL-8 production goes from 20 to 4,000 pg/mL blood in response to MOI 0.1 rBCG-S1PT in 24 h. It is also clear that individual donors can be differently sensitive to the enhanced effect of rBCG-S1PT, as for instance in the case of the superior IL-1β induction shown in Figure 1, while this effect was not evident when examining a larger cohort (Figure 2B). This would suggest the need of personalized testing for patients undergoing BCG therapy for bladder cancer.

The results shown in Figure 2 were obtained with blood cells from 34 donors that were vaccinated with BCG at birth. In order to examine whether the differential responsiveness to rBCG-S1PT vs. WT-BCG depended on previous vaccination the response to BCG was assessed in a very limited group of non-vaccinated donors (*n* = 5), for the reason that BCG vaccination is compulsory in Brazil since 1967 and non-vaccinated individuals are rare. The results in Supplementary Figure 2 show that non-vaccinated donors also respond to rBCG-S1PT with a higher production of IL-8, IL-6, and IL-10, as shown for vaccinated donors. Notably, the absolute activation level is about 5–10-fold lower in non-vaccinated donors (both for BCG strains and for LPS), but the activation induced by rBCG-S1PT reaches a level that is within the same range of the response of vaccinated donors to WT-BCG (IL-8: 200–380 ng/ml with rBCG-S1PT in non-vaccinated donors vs. 50–3,000 ng/ml with WT-BCG in vaccinated donors; IL-6: 0–48 ng/ml with rBCG-S1PT in non-vaccinated vs. 0–190 ng/ml with WT-BCG in vaccinated donors; IL-10: 20–180 pg/ml with rBCG-S1PT in non-vaccinated donors vs. 0–200 pg/ml with WT-BCG in vaccinated donors) (Supplementary Figure 2).

### rBCG-S1PT Induces Activation of Human Blood CD4^+^ T Cells

**Figure 3:**
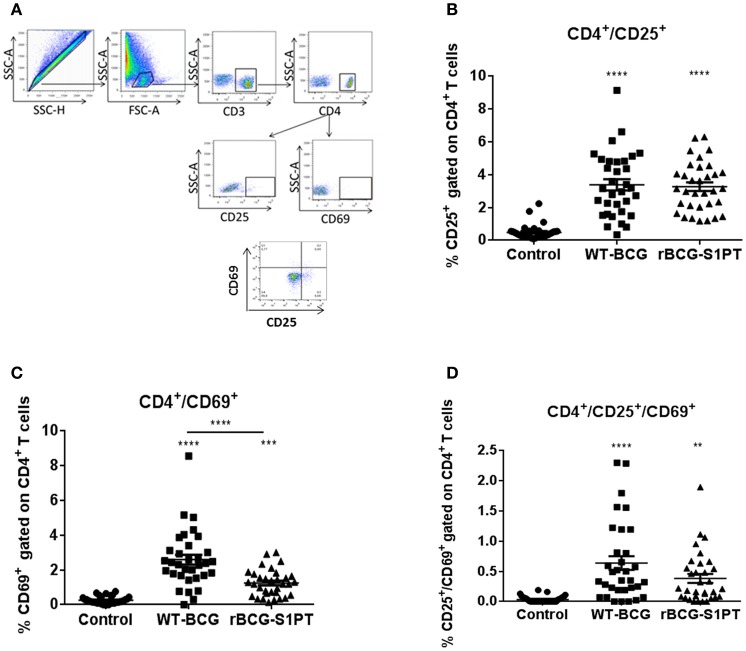
CD4^+^ T cell activation in blood cells from healthy adults stimulated with WT-BCG or rBCG-S1PT. Blood cells were stimulated with WT-BCG or rBCG-S1PT for 48 h and analyzed phenotypically by flow cytometry. **(A)** gating strategy; **(B)** percentage of CD4^+^/CD25^+^ cells; **(C)** percentage of CD4^+^/CD69^+^ cells; **(D)** percentage of CD4^+^/CD69^+^/CD25^+^ cells. Negative controls are non-stimulated cells. ****p < 0.0005, ***p < 0.001, and **p < 0.01 vs. negative controls.

To investigate whether rBCG-S1PT is able to activate T cells, we examined the expression of CD25 and CD69 on human blood CD4^+^ T cells upon exposure to rBCG-S1PT, in comparison to WT-BCG. Figure 3A shows the gating strategy for cytofluorimetric marker detection. Both BCG strains significantly enhanced the expression of CD25 molecules on CD4^+^ T cells (Figure 3B), implying a comparable capacity to activate CD4^+^ T cells. We also evaluated the expression of the early activation marker CD69. Both WT-BCG and rBCG-S1PT could enhance CD69 expression on CD4^+^ T cells, although rBCG-S1PT was not as efficient as WT-BCG (Figure 3C). When assessing the number of CD4^+^ T cells co-expressing CD25 and CD69, both BCG strains efficiently and comparably enhanced the number of activated cells (Figure 3D).

### rBCG-S1PT Stimulation of Human Blood Cells Induces Tumor Cytotoxicity

**Figure 4:**
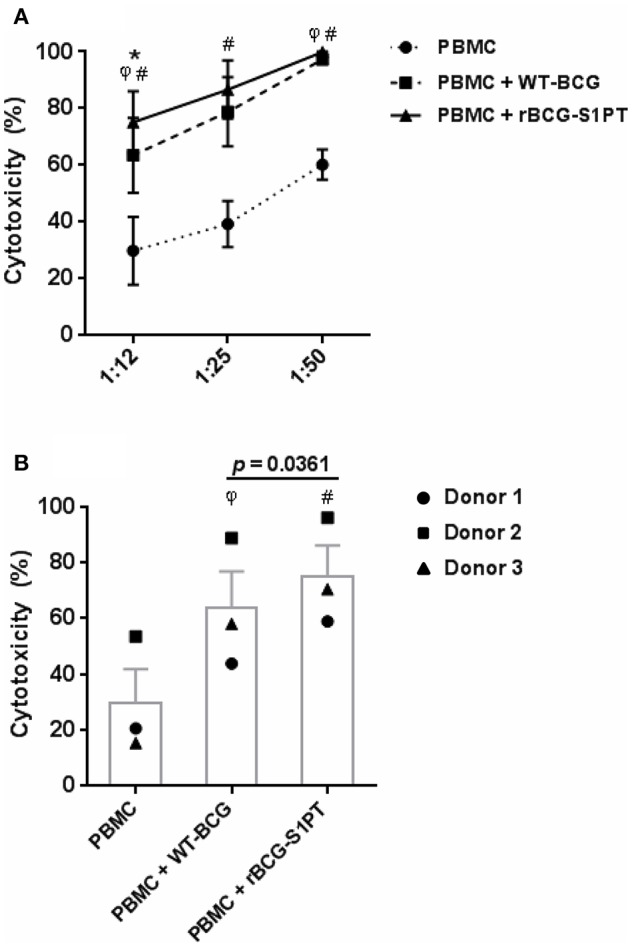
Tumor cytotoxicity of human blood mononuclear cells activated with WT-BCG or rBCG-S1PT. **(A)** Blood mononuclear cells were incubated for 24 h with MB49-GFP bladder tumor cells at different effector to target ratios (12.5:1, 25:1, 50:1) in the absence or in the presence of WT-BCG or rBCG-S1PT at MOI 0.1. Tumor cytotoxicity was assessed as % reduction of cell number *vs*. control cells incubated without PBMC and without BCG and expressed as mean ± SEM of three donors. **(B)** Data from 3 individual donors are presented at the effector to target ratio of 12.5:1, two that were vaccinated with BCG (1 and 3) and one that was not vaccinated (2). Statistical significance was assessed by Student's *t*-test. ^φ^*p* < 0.05, WT-BCG vs. control PBMC; #*p* < 0.05, rBCG-S1PT vs. control PBMC; *p < 0.05, rBCG-S1PT vs. WT-BCG. In panel B, the *p* value for the difference between BCG groups is indicated over the bar.

To investigate whether rBCG-S1PT can activate the anti-tumor activity of human leukocytes, we have set up an *in vitro* assay that measures the global cytotoxic effect, comprising both cytolysis (cell death) and cytostasis (block/decrease of cell proliferation). The assay is based on the assessment of the number of living tumor cells present in culture after incubation with effector leukocytes. To mimic *in vivo* conditions, leukocytes were co-cultured with cells from a bladder cancer cell line in the presence of BCG. In the conditions used, BCG had no direct cytotoxic effect on tumor cells (not shown). As shown in Figure 4, PBMC of three different donors showed a measurable spontaneous cytotoxic effect for tumor cells, which was significantly increased by both WT-BCG and rBCG-S1PT (Figure 4A). For each donor, the effect of recombinant BCG was higher than that induced by the wild type strain and is particularly evident at the effector to target ratio of 12.5:1 (Figure 4B). At higher effector to target ratios (25:1, 50:1) the cytotoxicity was nearly 100% for all donors with both BCG strains (Figure 4A).

## Discussion

We provide evidence that rBCG-S1PT is efficient in the activation of human CD4^+^ lymphocytes and of innate/inflammatory immune response, the latter being superior to that induced by WT-BCG, likely relying on the potent non-specific adjuvant capacity of the expressed detoxified S1PT (20). Previous data showing the improved performance of rBCG-S1PT in a mouse model of bladder cancer (25, 26) are supported by data in this study, showing a peculiar activation profile induced by the recombinant strain that is suggestive of a more effective initiation and regulation of immune reactivity.

Our results show that stimulation of human blood cells *in vitro* with both rBCG-S1PT and WT-BCG induce the production of inflammatory cytokines, essential for the induction of non-specific innate immune activation, within 24 h. While levels of the classical inflammatory cytokines IL-1β and TNF-α are enhanced in a comparable fashion by both strains, production of IL-8 and IL-6 is significantly higher in rBCG-S1PT-stimulated cells. Urinary levels of IL-8, IL-6, and also IL-10, are important markers to evaluate the efficacy of BCG instillations in the treatment of NMIBC (30). Urinary IL-8 has been considered a potential prognostic factor for tumor recurrence and progression following BCG therapy (31), while decreased IL-6 serum levels in patients with bladder cancer correlate with the appearance of myeloid-derived suppressor cells and poor prognosis (32). The capacity of PT to induce IL-6 production in mice (33, 34) may explain the finding that human blood cells produce higher IL-6 levels when stimulated with rBCG-S1PT in comparison to WT-BCG.

In the murine bladder cancer model, treatment with rBCG-S1PT induced an increase in IL-10 mRNA and in the survival time of the animals (25). Here we demonstrated that rBCG-S1PT induces a significantly higher production of IL-10, in parallel to the favorable markers IL-8 and IL-6, as compared to WT-BCG, also in human blood leukocytes. The higher production of IL-10 induced by rBCG-S1PT as compared to WT-BCG reveals a tighter control of the Th1-dependent inflammatory response, which may result in an effective induction of response in the absence of significant inflammatory side effects. Thus, the enhanced IL-10 production should be considered as a mechanism of protection against tissue injury and pathological processes associated with Th1 and inflammatory cytokines (35, 36).

The overproduction of IL-6 and IL-8 in combination with increased IL-10 and stable production of IL-1β and TNF-α suggests that rBCG-S1PT establishes an efficient compensatory mechanism to control excessive inflammation.

IFN-γ production was induced by both BCG strains in about one third of donors, while all others were unreactive. This is similar to the production of TNF-α, and suggests a significant heterogeneity in individual responsiveness to BCG, relative to these key inflammatory cytokines, which may imply different outcomes in terms of therapeutic efficacy. Other cytokines such as IL-2 and IL-17 were not significantly induced by either BCG strain. IL-17 was measurably produced by non-stimulated blood cells in about two thirds of the donors, and exposure to BCG did not increase its production nor the number of cytokine-producing donors. Also in the case of IL-2, two subpopulations of donors, responders and non-responders, could be identified. It is notable that in the case of IL-2 the vast majority of donors was able to respond to rBCG-S1PT, while only two thirds of donors could respond to WT-BCG. The Th-related cytokines IL-12 and IL-4 were not produced in response to either BCG strain, suggesting that the major effects of BCG are on innate immunity and inflammatory responses, without a significant direct effect on the later stages of adaptive immune response.

Immunological evaluation of patients with NMIBC show that BCG treatment induces predominantly CD4^+^ activation (7). Indeed, *in vitro* exposure of human blood cells to BCG strains induces activation of CD4^+^ T cells. Our results show that *in vitro* stimulation of blood cells induced an increase in the number of CD4^+^/CD25^+^ T cells, which is comparable between WT-BCG and rBCG-S1PT. This is in agreement with previous findings that show induction of CD25 (IL-2Rα, a marker of T cell activation) on lymphocytes following infection with mycobacteria *in vivo* and *in vitro* (37, 38). Stimulation of blood cells with rBCG-S1PT or WT-BCG also induced a significant increase in the early activation marker CD69 in CD4^+^ T cells, an increase that has been correlated with immune sensitization to mycobacterial antigens (39). In the present study, both WT-BCG and rBCG-S1PT induced CD69 expression on blood CD4^+^ T cells. The higher increase induced by WT-BCG suggests a more pronounced early activation. However, fully activated CD4^+^ T cells co-expressing CD25 and CD69 were equally induced by rBCG-S1PT and WT-BCG, implying an essentially similar T cell activation profile.

Enhanced IL-8 and IL-6 production in response to rBCG-S1PT implies improved recruitment and stimulation of blood-derived immune cells. On the other hand, it is notable that there is no significant difference in the induction of IL-1β and TNF-α, both endowed with potent inflammatory properties but important for immune stimulation. The stable production of these two factors suggests that, while immune stimulation is maintained at the same level as with WT-BCG, rBCG-S1PT does not increase detrimental inflammatory effects.

It has been shown that BCG-stimulated PBMCs induce death of tumor cell lines through a complex mechanism involving the activation of macrophages and CD4^+^ T cells, which leads to the generation of BCG-activated killer cells (BAK cells - CD8^+^ T cells) stimulated by IL-2 and IFN-γ (40). We investigated the ability of rBCG-S1PT to activate the direct cytotoxic activity of human leukocytes against tumor cells *in vitro*. Notably, while both WT-BCG and rBCG-S1PT significantly increased the cytotoxic capacity of leukocytes, the recombinant strain induced a higher effect on cells from all the tested donors. It will be important to further investigate the relative role of lymphocytes and monocytes (which also get potently activated by BCG *in vitro*; data not shown) in the cytotoxicity against MB49 cells.

Our study was mainly run with blood samples taken from BCG-vaccinated volunteers. It is interesting to note that, in a limited number of non-immunized donors, reactivity to the two BCG strains displayed the same profile as that described here for immunized donors, although the absolute levels of cytokines were lower. Indeed, there are suggestions in the literature that BCG vaccination may improve the efficacy of BCG immunotherapy in bladder cancer therapy, both in experimental models and in human patients (41). A pilot study is currently ongoing to assess the effect of BCG vaccination in bladder cancer patients (42).

Based on the data described here, we can conclude that human blood cells challenged with rBCG-S1PT in a realistic *in vitro* model show an improved immune activation profile in comparison to WT-BCG, in terms of cytokine production and tumor cytotoxicity. This evidence strengthens the results of improved bladder cancer treatment in a mouse model (25) and indicate rBCG-S1PT as an excellent candidate for a more effective immunotherapy of non-muscle-invasive bladder cancer.

## Ethics Statement

The study protocol was approved by the Comitê de Ética/Pesquisa Hospital Universitário/USP (CEP-HU/USP) 728.275.

## Author Contributions

DR, CG, IN, RB, WD, AT, DB, and LL: contributed to the design of the experiments, analyzed, and interpreted data. DR, CG, AP, ES, PC, and DB: performed the experiments. DR, CG, WD, AT, DB, and LL: wrote the paper.

### Conflict of Interest Statement

LL and IN have a patent application on the use of rBCG-S1PT in bladder cancer treatment. The remaining authors declare that the research was conducted in the absence of any commercial or financial relationships that could be construed as a potential conflict of interest.

## Supplementary Material

Supplementary Figure 1 Time-dependent production of cytokines by blood cells from healthy adults stimulated with WT-BCG or rBCG-S1PT. Blood cells were stimulated with WT-BCG or rBCG-S1PT and cytokines were measured with a CBA array at 24 and 48 h. Values represent the concentration of cytokines per mL blood. Negative controls are non-stimulated cells. Positive controls (cells stimulated with LPS) were: IL-6, 45.53 ± 8.40 ng/mL; TNF-α, 0.10 ± 0.01 ng/mL; IL-10, 123.56 ± 33.38 pg/mL. ^****^*p* < 0.0005, ^***^*p* < 0.001, ^**^*p* < 0.01, ^*^*p* < 0.05 vs. negative controls.

Supplementary Figure 2 Production of cytokines by blood cells from non-vaccinated and BCG-vaccinated healthy adults stimulated with WT-BCG or rBCG-S1PT. Blood cells from non-vaccinated donors (left panels) or donors vaccinated with BCG (right panels, same as in Figure 2 and Supplementary Figure 1) were stimulated with WT-BCG or rBCG-S1PT for 24 h. Cytokines were measured with a CBA array and expressed as concentration per mL blood. Negative controls are non-stimulated cells. Positive controls (cells stimulated with LPS) were: for non-vaccinated donors, IL-8, 46.86 ± 4.26 ng/mL; IL-6, 36.22 ± 8.10 ng/mL; IL-10, 8.72 ± 0.63 pg/mL; for vaccinated donors, IL-8, 133.40 ± 13.63 ng/mL; IL-6, 80.11 ± 12.30 ng/mL; IL-10, 95.04 ± 17.61 pg/mL; ^****^*p* < 0.0005, ^***^*p* < 0.001, and ^**^*p* < 0.01 vs. negative control or WT-BCG groups.

## Abbreviations

BCG: *Mycobacterium bovis* Bacille Calmette-Guérin
NMIBC: non-muscle invasive bladder cancer
PBMC: peripheral blood mononuclear cells
PT: pertussis toxin
rBCG-S1PT: recombinant BCG expressing the detoxified PT S1 unit
WT-BCG: wild type BCG.
