# Supplementary figures and images for: In vitro Evidence of Human Immune Responsiveness Shows the Improved Potential of a Recombinant BCG Strain for Bladder Cancer Treatment
Source: Front Immunol. 2019 Jun 26;10:1460. doi: 10.3389/fimmu.2019.01460 (PMC6607967; doi:10.3389/fimmu.2019.01460)

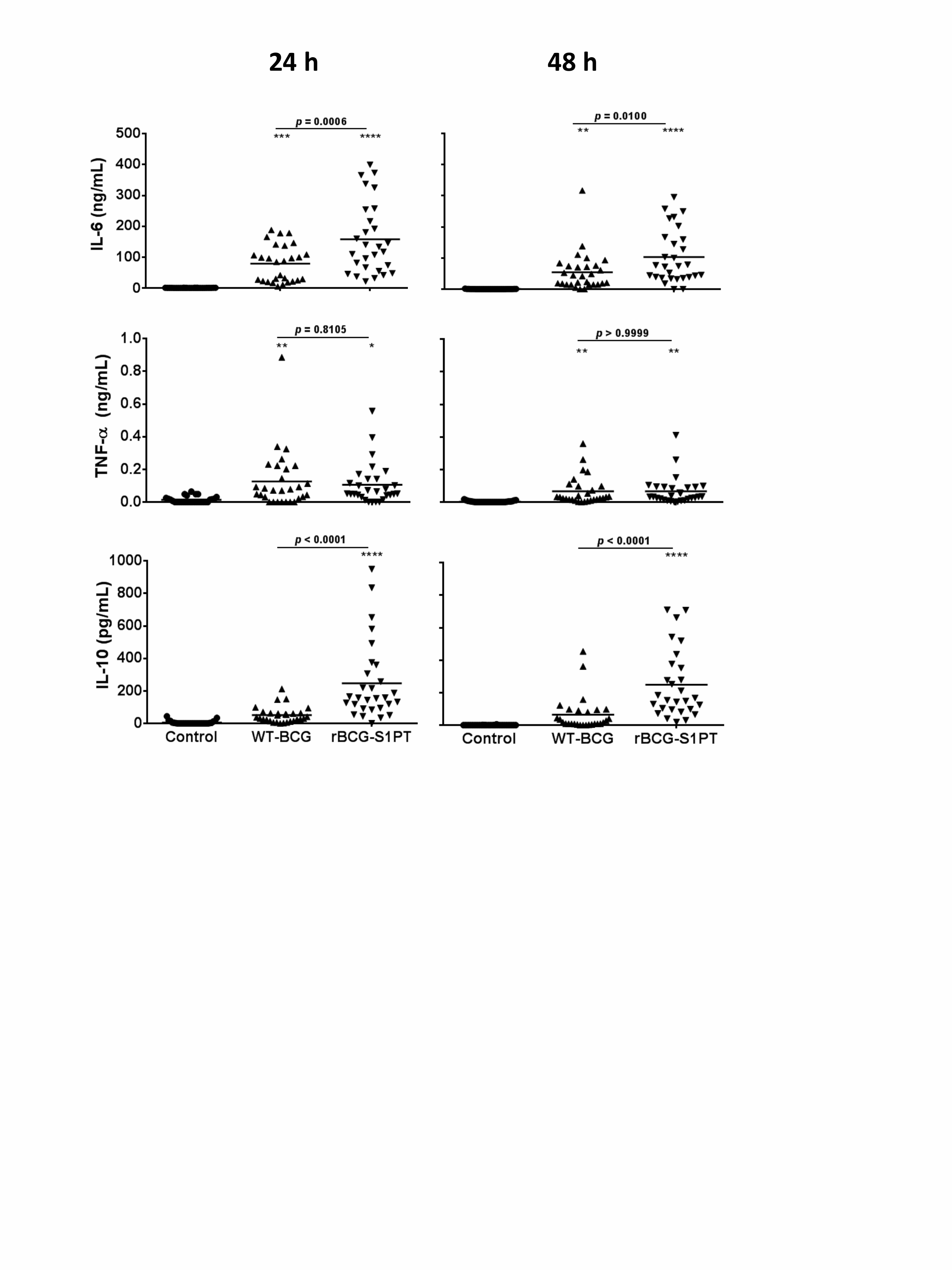

Supplement: Supplementary Figure 1 — Time-dependent production of cytokines by blood cells from healthy adults stimulated with WT-BCG or rBCG-S1PT. Blood cells were stimulated with WT-BCG or rBCG-S1PT and cytokines were measured with a CBA array at 24 and 48 h. Values represent the concentration of cytokines per mL blood. Negative controls are non-stimulated cells. Positive controls (cells stimulated with LPS) were: IL-6, 45.53 ± 8.40 ng/mL; TNF-α, 0.10 ± 0.01 ng/mL; IL-10, 123.56 ± 33.38 pg/mL. ****p < 0.0005, ***p < 0.001, **p < 0.01, *p < 0.05 vs. negative controls. [file Image_1.TIFF]

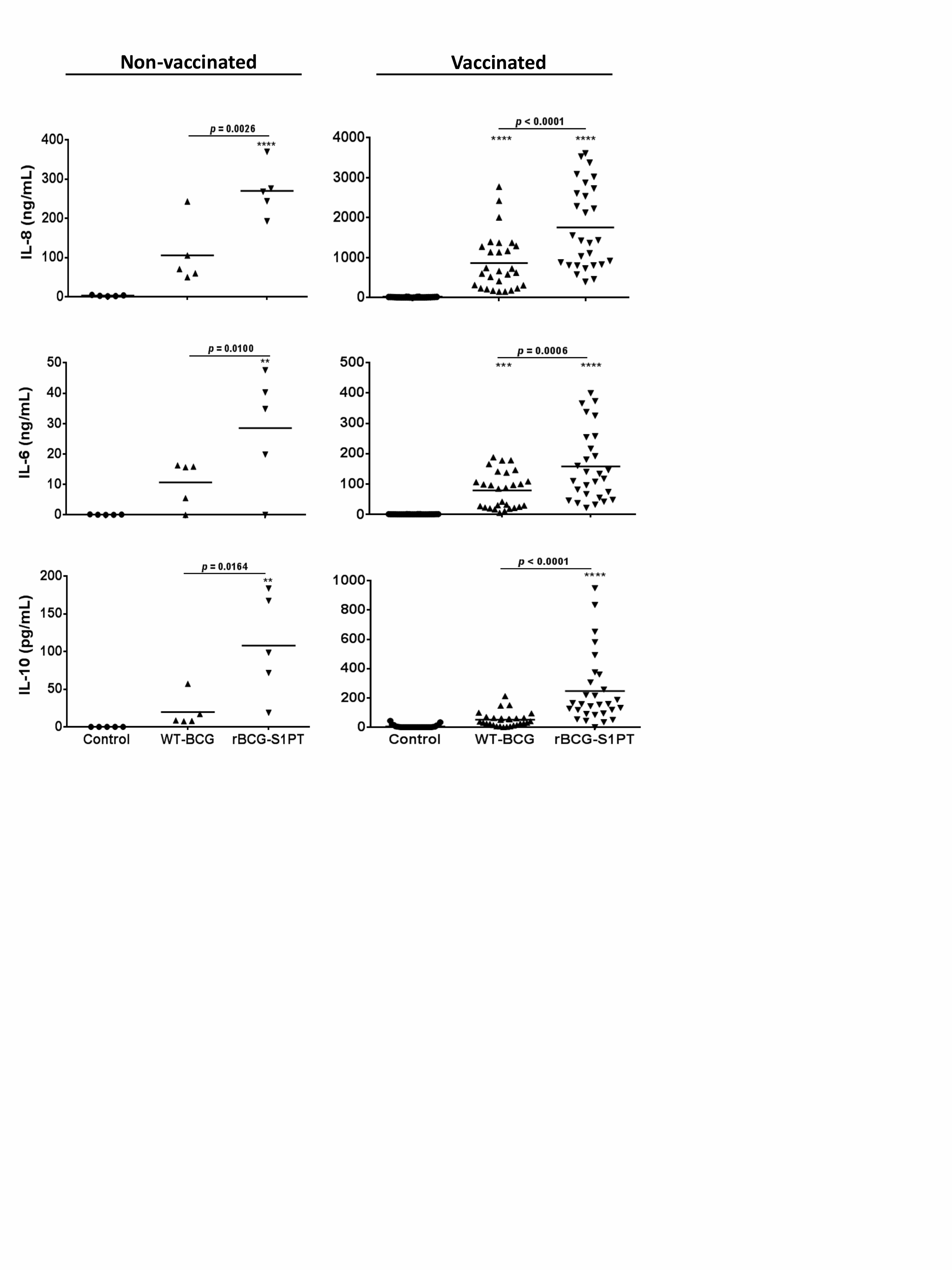

Supplement: Supplementary Figure 2 — Production of cytokines by blood cells from non-vaccinated and BCG-vaccinated healthy adults stimulated with WT-BCG or rBCG-S1PT. Blood cells from non-vaccinated donors (left panels) or donors vaccinated with BCG (right panels, same as in Figure 2 and Supplementary Figure 1) were stimulated with WT-BCG or rBCG-S1PT for 24 h. Cytokines were measured with a CBA array and expressed as concentration per mL blood. Negative controls are non-stimulated cells. Positive controls (cells stimulated with LPS) were: for non-vaccinated donors, IL-8, 46.86 ± 4.26 ng/mL; IL-6, 36.22 ± 8.10 ng/mL; IL-10, 8.72 ± 0.63 pg/mL; for vaccinated donors, IL-8, 133.40 ± 13.63 ng/mL; IL-6, 80.11 ± 12.30 ng/mL; IL-10, 95.04 ± 17.61 pg/mL; ****p < 0.0005, ***p < 0.001, and **p < 0.01 vs. negative control or WT-BCG groups. [file Image_2.TIFF]
